# β_1_-integrin controls IGF-1R internalization and intracellular signaling

**DOI:** 10.1016/j.jbc.2024.108021

**Published:** 2024-11-27

**Authors:** Niamh McDermott, Stephen O’Shea, Leonie Rieger, Orla T. Cox, Rosemary O’Connor

**Affiliations:** Cell Biology Laboratory, School of Biochemistry and Cell Biology, University College Cork, Cork, Ireland

**Keywords:** breast cancer, cancer, cell adhesion, insulin-like growth factor 1 receptor (IGF-1R), integrin, phosphorylation, receptor internalization, receptor structure-function, subcellular location, transformation

## Abstract

Cell adhesion-dependent phosphorylation of insulin-like growth factor 1 receptor (IGF-1R) on its C-terminal tail (CT) at Tyr^1250/1251^ promotes receptor internalization and Golgi accumulation. We previously proposed that this phosphorylation is associated with cell migration and cancer aggressiveness, distinguishing IGF-1R activity from that of insulin receptor, which lacks these tyrosines. Here, we further investigated how adhesion signaling influences IGF-1R location and activity in migratory cancer cells and R- fibroblasts. We observed that IGF-1R, in triple-negative breast cancer tissues, is predominantly intracellular and dispersed from the plasma membrane compared with nontumor tissue. Datasets from basal-like breast cancer patients indicated a strong, positive correlation between IGF-1R protein expression and that of β_1_-integrin (ITGB1). In triple-negative breast cancer cells with high ITGB1 expression, suppressing ITGB1 enhanced IGF-1R stability and its retention at the plasma membrane, and reduced IGF-1R internalization during cell adhesion. In R- fibroblasts, we observed reduced IGF-1R autophosphorylation and Golgi accumulation when ITGB1 was suppressed. The stability of a Tyr^1250/1251^Phe (FF) IGF-1R mutant was less affected by ITGB1 suppression, indicating that Tyr^1250/1251^ phosphorylation is required for ITGB1-enhanced receptor internalization. Furthermore, a Tyr^1250/1251^Glu (EE) IGF-1R mutant exhibited a gain of cell migration and colony formation potential compared to WT IGF-1R or FF mutant. Tyr^1250/1251^ resides within the CT ^1248^SFYYS^1252^ motif, which engages the IGF-1R kinase domain. *In silico*, we investigated how mutation of these tyrosines may alter ^1248^SFYYS^1252^ conformation, dictating trajectory of the distal CT. We conclude that Tyr^1250/1251^ phosphorylation confers IGF-1R with unique protumorigenic signaling in a manner that is enhanced by ITGB1.

The insulin-like growth factor 1 receptor (IGF-1R) and its ligands (IGF-1 and IGF-2) are essential for cell, organ, and organismal growth (1), and are linked to cancer risk, cancer progression, and inflammation (2, 3, 4, 5). Though highly promising as a druggable candidate in preclinical cancer studies, therapies targeting the IGF-1R have failed to show clinical efficacy in several malignancies (6, 7, 8, 9, 10). Surprisingly, an anti-IGF-1R monoclonal antibody, intended as an anticancer treatment, has been repurposed, and approved, as a therapy for the inflammatory condition, thyroid eye disease (11). Although extensively deliberated (12, 13, 14, 15), it remains unresolved why IGF-1R inhibition has been largely ineffective in cancer, yet was successful in an inflammatory context (16, 17, 18). However, it has become increasingly apparent that context-dependent signals between IGF-1R and other pathways are important regulators of IGF-1R signaling output and its potential for clinical targeting (19, 20).

Cooperation between IGF and adhesion signaling in cancer and other proliferating or migrating cells is well-documented (21, 22, 23, 24, 25, 26, 27). In particular, engagement with integrins, namely β_1_-(ITGB1) or β_3_-integrin (ITGB3), has been shown to enhance IGF-1R signaling in cancer cell models, fibroblasts, and endothelial cells (28, 29, 30, 31). In a recent study, we demonstrated that cell adhesion regulates phosphorylation of IGF-1R on Tyr^1250/1251^, and that this is required for receptor internalization and subsequent accumulation at the Golgi apparatus in migratory cells (32). Like in other receptor tyrosine kinases, the IGF-1R C-terminal tail (CT) is proposed to regulate receptor activity (33, 34), and potentially elicit context-specific signals depending on its phosphorylation status or potential interactions with other proteins (30, 35, 36, 37, 38, 39). Deletion of the entire CT impairs cellular transformation (40), and mutation of Tyr^1250/1251^ to nonphosphorylatable phenylalanines is sufficient to abrogate cell survival, transforming, and motility signals from the receptor (41, 42, 43, 44). Phosphorylation of these tyrosines is also more evident in migratory than less migratory cells (32), further suggesting that this phospho-site is critical for integrating or mediating the adhesion-dependent cell migration and protransformation functions of IGF-1R.

In this study, we investigated how cell adhesion controls IGF-1R location and activity in cancer cells and fibroblasts. In agreement with our previous observations in cell lines, we confirmed, by immunofluorescence, that IGF-1R is located less at the plasma membrane in triple-negative breast cancer (TNBC) tumor tissue than in nontumor tissue. Next, based on our and others’ previous observations relating to the IGF-1R/adhesion signaling axis (22, 23, 24, 25, 28, 29, 30, 31), we focused on the relationship between IGF-1R and the integrin subunits, ITGB1 and ITGB3. Unlike with ITGB3, we observed a strong, positive correlation between IGF-1R and ITGB1 protein expression in basal-like breast cancer patients. In migratory cancer cells, which express high levels of ITGB1, suppression of ITGB1 increased IGF-1R stability and its retention on the plasma membrane during cell adhesion. Moreover, ITGB1 suppression decreased signaling output from WT IGF-1R and increased the stability of a Tyr^1250/1251^Glu phosphomimetic (EE) IGF-1R mutant and WT IGF-1R, but not a nonphosphorylatable Tyr^1250/1251^Phe (FF) mutant. R- cells stably expressing the EE mutant exhibited Golgi-located receptor, as well as enhanced migratory and anchorage-independent growth capabilities relative to WT IGF-1R, indicating that phosphorylation of this site may promote a more transformed cellular phenotype. Finally, structural analysis of WT IGF-1R and FF/EE mutants using the DynaMut2 tool indicated that mutation of the Tyr^1250/1251^ site may alter IGF-1R ^1248^SFYYS^1252^ motif conformation, thereby dictating CT trajectory. This may explain the enhanced and impaired activities of EE and FF IGF-1R mutants, respectively.

Overall, these findings indicate that ITGB1-dependent phosphorylation and trafficking of the IGF-1R enhances its signaling output, which may be critical in promoting an aggressive cancer phenotype. This also distinguishes IGF-1R signaling from insulin receptor (IR) signaling because these twin tyrosine residues are not present in human IR (45, 46).

## Results

### Intracellular IGF-1R in cancer cell lines and TNBC tissues

**Figure 1:**
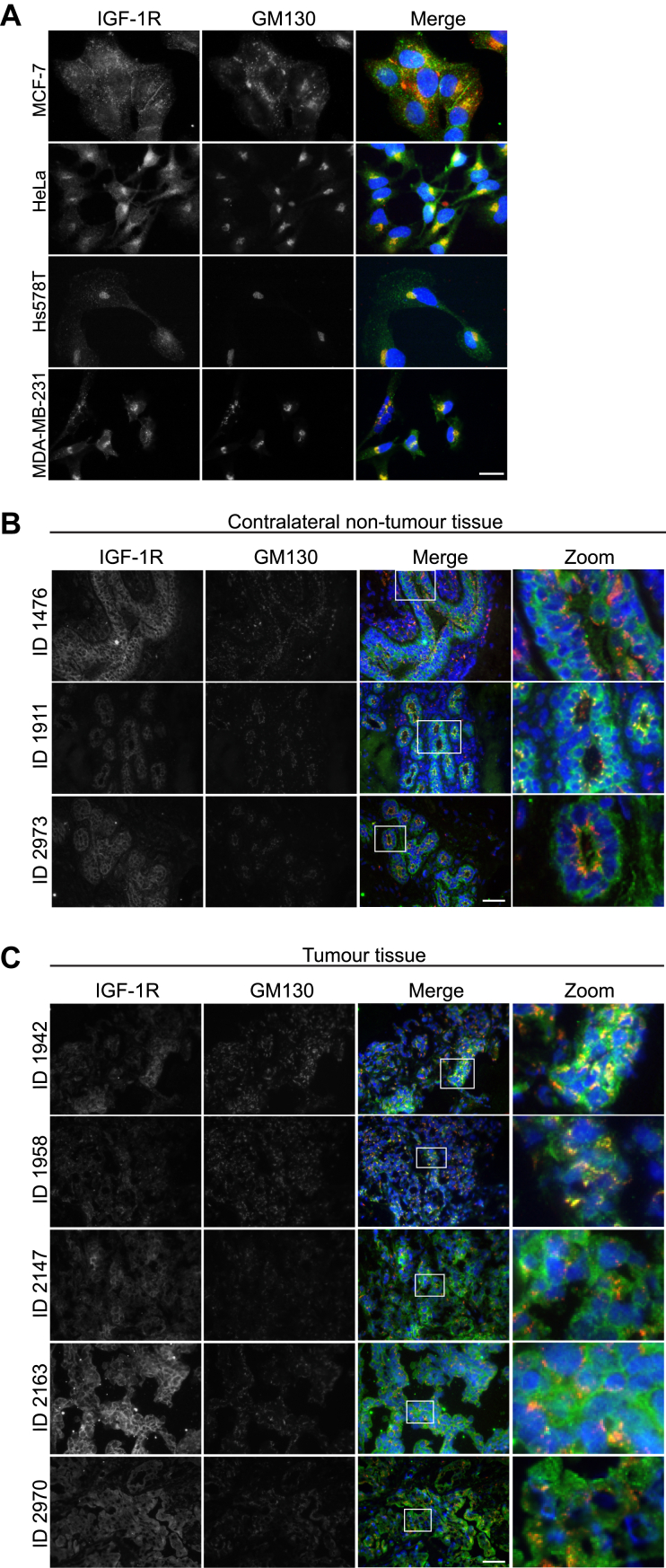
**The IGF-1R isdispersedfrom the plasma membrane and dispersed throughout the cytoplasm in TNBC.***A*, immunofluorescence images (representative of n = 3) of MCF-7, HeLa, Hs578T, and MDA-MB-231 cells. Cells were seeded on glass coverslips and left to adhere for 24 h. Cells were stained for IGF-1R (*green*) and the Golgi marker, GM130 (*red*), with Hoechst nuclear stain in *blue*. IGF-1R/GM130 colocalization is indicated by *yellow* signal. Magnification = 100×. The scale bars represent 20 μm. *B*, immunofluorescence of fresh frozen contralateral, nontumor tissue sections from TNBC patients. Images show IGF-1R in *green*, GM130 in *red*, and Hoechst nuclear stain in *blue*. *C*, immunofluorescence of fresh frozen tumor tissue sections from TNBC patients. Images show IGF-1R in *green*, GM130 in *red*, and Hoechst nuclear stain in *blue*. Magnification = 40×. The scale bars represent 200 μm. IGF-1R, insulin-like growth factor 1 receptor; TNBC, triple-negative breast cancer.

In our previous study (32), we reported that the presence of IGF-1R at the Golgi apparatus was more evident in migratory than nonmigratory breast cancer cell lines. Here, we confirmed these findings. Colocalization of IGF-1R with the Golgi marker, GM130, was evident in two TNBC cell lines, Hs578T and MDA-MB-231. We also expanded the study to include another migratory cell line, HeLa cervical carcinoma cells, in which we also observed IGF-1R at the Golgi (Fig. 1*A*). Conversely, in hormone-responsive MCF-7 luminal breast cancer cells, which are less migratory, IGF-1R was more evident at the plasma membrane and at cell-cell and cell-matrix contact points (Fig. 1*A*), as previously documented (32, 47).

To further investigate the physiological relevance of IGF-1R subcellular location in TNBC, we carried out immunofluorescence staining for IGF-1R and GM130 on fresh frozen tissue sections from TNBC patients (n = 10). The sections were derived from either contralateral, nontumor breast cancer tissue or from tumor tissue. The IGF-1R was present at the plasma membrane and at intraepithelial junctions in normal breast tissue epithelium, with only little costaining with the Golgi observed (Fig. 1*B*). In contrast, the IGF-1R exhibited a more dispersed staining pattern in all tumor tissue sections, with evidence of cytoplasmic and Golgi localization. This staining pattern accompanied a compromised tissue architecture (Fig. 1*C*). A similar pattern of IGF-1R staining in invasive breast cancer tissues is reported in the Human Protein Atlas database (Fig. S1*B*).

Taken together, these data indicate that IGF-1R dispersal from the plasma membrane and intraepithelial junctions is associated with loss of overall tissue architecture, and that IGF-1R is predominantly intracellular in TNBC tissues. This suggests that colocation with the Golgi apparatus may be a feature of IGF-1R activity in migratory cancer cells.

### **β**_1_-integrin suppression stabilizes IGF-1R protein levels in migratory cancer cells

**Figure 2:**
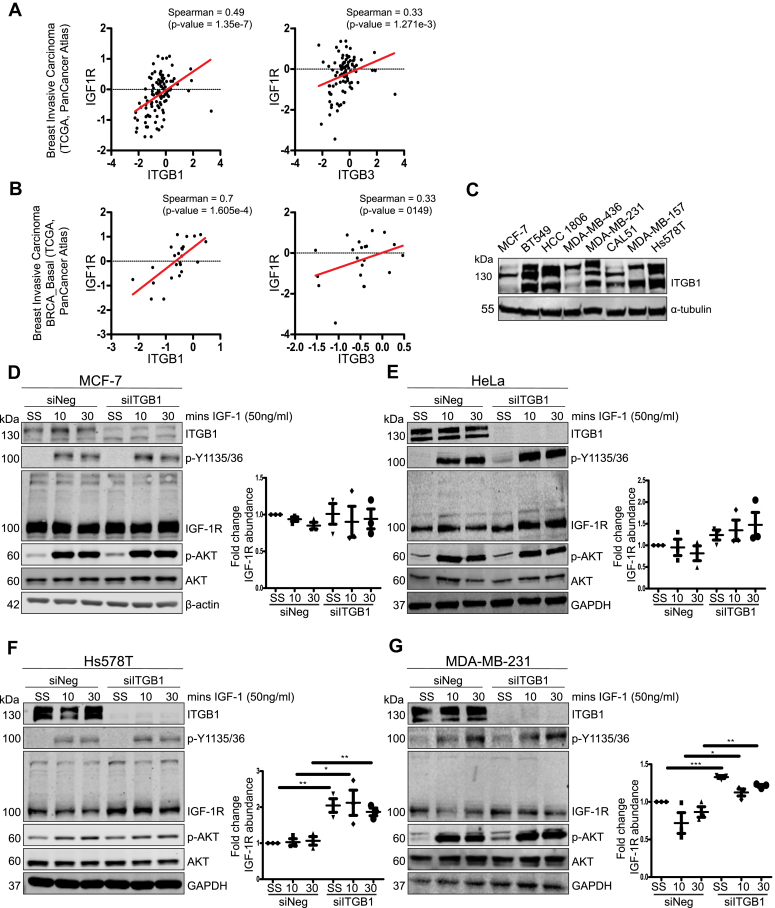
**ITGB1 is highly expressed in migratory cancer cells and is required for IGF-1R turnover.***A*, correlation of ITGB1 (*left*) or ITGB3 (*right*) protein expression with IGF-1R expression using data from TCGA, PanCancer Atlas—Breast Invasive Carcinoma dataset (n = 105). Relative protein expression levels were measured by mass spectrometry by the Clinical Proteomic Tumor Analysis Consortium (CPTAC), represented as arbitrary units (A.U.) on a distribution. *B*, correlation of ITGB1 (*left*) or ITGB3 (*right*) protein expression with IGF-1R expression using data from TCGA, PanCancer Atlas—Breast Invasive Carcinoma: Basal subtype datasets (IGF1RvITGB1, n = 24; IGF1RvITGB3, n = 21). Relative protein expression levels measured by mass spectrometry carried out by CPTAC—represented as arbitrary units on a distribution. *C*, western blot showing ITGB1 expression in a panel of breast cancer cell lines. *D*–*G*, western blot analysis of (*D*) MCF-7, (*E*) HeLa, (*F*) Hs578T, and (*G*) MDA-MB-231 cells transfected with either siNeg or siITGB1 siRNA. Cells were serum starved for 4 h and then stimulated with 50 ng/ml IGF-1 for either 10 min or 30 min. For (*D*–*G*) densitometry quantification is presented as the average fold change ± SEM in IGF-1R protein levels relative to the unstimulated control sample (set to 1). Representative of n = 3 independent experiments. Significance was calculated using an unpaired, two-tailed Student’s *t* test. Statistical significance was determined as *p*-value <0.05. Graded *p*-values are represented as follows: ∗*p* < 0.05, ∗∗*p* < 0.005, and ∗∗∗*p* < 0.0005. IGF-1R, insulin-like growth factor 1 receptor; ITGB1, β_1_-integrin; TCGA, The Cancer Genome Atlas.

IGF-1R accumulation at the Golgi apparatus is promoted by cell adhesion-dependent phosphorylation of Tyr^1250/1251^ in the IGF-1R CT (32). Therefore, we next asked how cell adhesion molecules influence IGF-1R activity in cancer. Using cBioPortal (48), we first investigated the relationship between the IGF-1R and two core adhesion molecules known to associate with the IGF-1R, ITGB1 and ITGB3 (28, 29, 30, 31). Using the The Cancer Genome Atlas (TCGA) Pan Cancer Atlas—Breast Invasive Carcinoma dataset (49) (n = 105), we found a moderate, positive correlation between IGF-1R and ITGB1 protein expression (Spearman = 0.49, *p* = 1.35 × 10^−7^), and a weak, positive correlation between IGF-1R and ITGB3 protein expression (Spearman = 0.33, *p* = 1.271 × 10^−3^) (Fig. 2*A*). Relative protein expression levels were measured by mass spectrometry carried out by the Clinical Proteomic Tumor Analysis Consortium and are represented as arbitrary units on a distribution. To further explore these relationships, a cohort of basal-like breast cancer patients (IGF1RvITGB1, n = 24; IGF1RvITGB3, n = 21) from the TCGA Pan Cancer Atlas—Breast Invasive Carcinoma dataset was further analyzed (because this breast cancer subtype has similar features to TNBC (50)). Here, we observed a strong, positive correlation between IGF-1R and ITGB1 protein expression (Spearman = 0.7, *p* = 1.605 × 10^−4^); however, the weak, positive correlation between IGF-1R and ITGB3 protein expression was not statistically significant (Spearman = 0.33, *p* = 0.149) (Fig. 2*B*).

Following this, we focused our study on how ITGB1 expression affects IGF-1R activity in migratory cancer cells. First, we assessed ITGB1 protein expression in a panel of breast cancer cell lines (Fig. 2*C*) and also in migratory HeLa and fibroblast cell lines (Fig. S2*A*). We observed that, in TNBC cell lines (*e.g.* BT549, MDA-MB-231, and Hs578T), ITGB1 was highly expressed, (and highly glycosylated as indicated by the presence of several higher molecular weight bands), relative to its expression in MCF-7 cells (Fig. 2*C*). It was also apparent that ITGB1 protein expression coincided with IGF-1R accumulation at the Golgi apparatus in Hs578T, MDA-MB-231, and HeLa cells (Fig. 1*A*) (32).

To further investigate the relationship between ITGB1 protein expression and IGF-1R activity, we transiently suppressed ITGB1 in MCF-7, Hs578T, MDA-MB-231, and HeLa cells by transfection with either siRNA targeting ITGB1 (siITGB1), or a nontargeting control (siNeg). IGF-1-induced phosphorylation of the IGF-1R activation loop residues, Tyr^1135/36^, and of the downstream signaling target, AKT, were unaffected by ITGB1 suppression in these epithelial cancer cell lines (Fig. 2, *D*–*G*). ITGB1 knockdown did not alter IGF-1R protein abundance in the less migratory MCF-7 cells (Fig. 2*D*); however, ITGB1 knockdown increased IGF-1R protein abundance in HeLa, Hs578T, and MDA-MB-231 cells, relative to the siNeg controls (Fig. 2, *E*–*G*). Although ITGB1 knockdown did not significantly alter IGF-1R protein levels in HeLa cells, it did significantly increase IGF-1R protein levels in Hs578T and MDA-MB-231 TNBC cells.

Overall, these data indicate that ITGB1 protein expression correlates with IGF-1R protein expression in breast cancer, particularly the basal-like subtype, and that high ITGB1 expression coincides with intracellular/Golgi-localized IGF-1R in migratory cells. Furthermore, loss of ITGB1 stabilizes IGF-1R protein levels, which may indicate that ITGB1 is required for IGF-1R protein turnover in migratory cancer cells.

### **β**_1_-integrin influences IGF-1R subcellular location in adherent cells and is required for IGF-1R internalization in actively adhering cells

**Figure 3:**
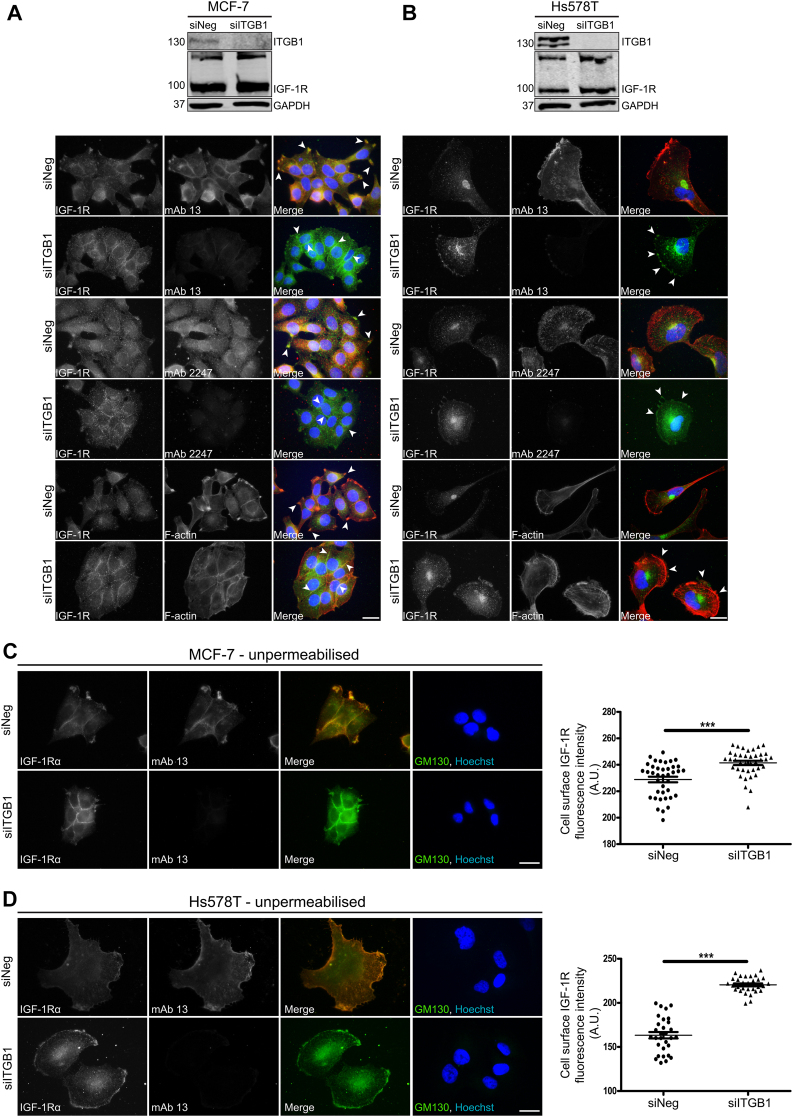
**ITGB1 suppression redistributes IGF-1R to the plasma membrane in adherent cells and increases IGF-1R presence at the cell surface.** Immunofluorescence staining of (*A*) MCF-7 cells, or (*B*) Hs578T cells transfected with siNeg or siITGB1 (representative of n = 3 experiments). Cells were seeded on glass coverslips and left to adhere for 24 h prior to fixation for immunofluorescence. IGF-1Rβ stained in *green*, ITGB1 (active, mAb 2247), ITGB1 (inactive, mAb 13), or F-actin (phalloidin) is stained in *red*, and Hoechst nuclear stain is shown in *blue*. The associated western blots confirm ITGB1 suppression. Unpermeabilized immunofluorescence staining of (*C*) MCF-7 cells, or (*D*) Hs578T cells transfected with siNeg or siITGB1. Cells were seeded on glass coverslips and left to adhere for 24 h prior to fixation for immunofluorescence. Cells were left unpermeabilized prior to blocking and staining. IGF-1Rα chain stained in *green*, and ITGB1 (inactive, mAb 13) is stained in *red*. Cells stained with the Golgi marker, GM130, in *green* as a permeabilization control with Hoechst nuclear stain also included to show presence of cells. Cell surface IGF-1R fluorescence intensity was quantified (n = 30 cells per condition) using ImageJ software and is represented as arbitrary units (A.U.) on a scatter plot. Significance was calculated using unpaired, two-tailed Student’s *t* test. Statistical significance was determined as *p*-value <0.05 and represented as follows: ∗∗∗*p* < 0.0001. Magnification = 100×. The scale bar represents 20 μm. IGF-1R, insulin-like growth factor 1 receptor; ITGB1, β_1_-integrin

As we observed that IGF-1R protein levels were influenced by ITGB1 expression, we next asked how transient suppression of ITGB1 affects IGF-1R subcellular location and trafficking in adherent, MCF-7 (low ITGB1 expression) *versus* TNBC Hs578T cells (high ITGB1 expression). We observed that, in siNeg control MCF-7 cells, the IGF-1R colocated primarily at cell-matrix contact points with the inactive form of ITGB1 (mAb 13), whereas active ITGB1 (mAb 2247) was primarily internalized and dispersed throughout the cell (Fig. 3*A*). Suppression of ITGB1 in MCF-7 cells caused loss of these cell-matrix contact points, which was accompanied by redistribution of IGF-1R from these regions to points of cell-cell contact (Fig. 3*A*). In siNeg control Hs578T cells, the IGF-1R was primarily located at a perinuclear compartment (Fig. 3*B*), consistent with Golgi location (Fig. 1*A*); however, suppression of ITGB1 in these cells increased IGF-1R presence at the cell periphery (Fig. 3*B*). To determine whether this staining at the cell periphery was indicative of surface IGF-1R expression, we repeated these experiments in unpermeabilized cells. In agreement with earlier observations, we observed a significant increase in IGF-1R fluorescence signal intensity on the surface of MCF-7 and Hs578T cells following ITGB1 suppression relative to their respective controls (Fig. 3, *C* and *D*), with the difference between the means being greater for Hs578T cells.

In Hs578T cells, the staining pattern of active and inactive ITGB1 included a pool at a perinuclear compartment, similar to IGF-1R. Notably, inactive ITGB1 has been shown to undergo retrograde trafficking to the Golgi (51). To determine whether ITGB1 and IGF-1R cotraffic to the Golgi apparatus, we transfected Hs578T cells with a fluorescent Golgi-localized construct, GalT-EGFP, and immunostained for either IGF-1R, active ITGB1 (mAb 2247), or inactive ITGB1 (mAb 13). We did not observe colocalization of the active or inactive forms of ITGB1 with GalT-EGFP in Hs578T cells. Only IGF-1R staining showed strong colocalization with GalT-EGFP (Fig. S3*A*). Furthermore, in Hs578T cells, the IGF-1R staining pattern was highly concentrated at the Golgi, whereas the ITGB1 staining pattern was predominantly dispersed throughout the cell and along the plasma membrane. It is possible that some ITGB1 accumulates at the Golgi with the IGF-1R; however, we could not detect this due to the weak signal intensity of ITGB1 relative to the IGF-1R and GalT-EGFP.

**Figure 4:**
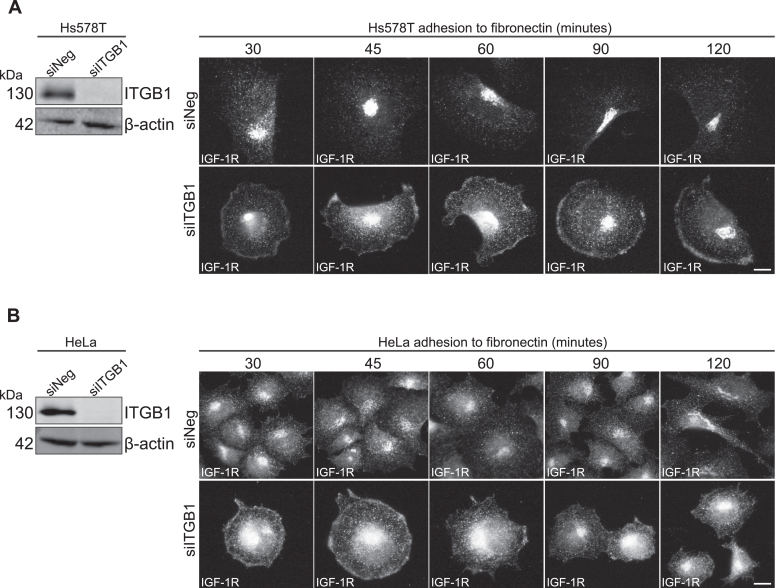
**ITGB1 delays IGF-1R internalization in actively adhering cells.** Immunofluorescence staining of (*A*) Hs578T cells and (*B*) HeLa cells transfected with siNeg or siITGB1. Cells were seeded 48 h after transfection on fibronectin-coated coverslips and left to adhere for the indicated time points prior to fixation for immunofluorescence. Cells were stained for IGF-1R (*gray scale*). For all images, magnification = 100×, the scale bars represent 20 μm. IGF-1R, insulin-like growth factor 1 receptor; ITGB1, β_1_-integrin.

To further test how ITGB1 affects IGF-1R activity, we asked whether ITGB1 influences IGF-1R subcellular location in actively adhering cancer cells. Hs578T and HeLa cells (transfected with either siNeg or siITGB1) were allowed to adhere to plates coated with the ITGB1 ligand, fibronectin, for the indicated time points from 30 to 120 min. Cells were stained for IGF-1R, as well as vinculin and F-actin, to delineate focal adhesions and the cell periphery, respectively (Figs. 4, *A* and *B* and S4, *A* and *B*). For both Hs578T and HeLa siNeg control cells, at the earliest time point, the IGF-1R was already observed to be internalized and trafficking away from the plasma membrane (Figs. 4, *A* and *B* and S4, *A* and *B*). This was consistent across all five time points. Conversely, when ITGB1 was suppressed in Hs578T and HeLa cells, from the earliest time point (30 min), both cell types exhibited a rounded-up morphology, indicating inability to spread and adhere (Figs. 4, *A* and *B* and S4, *A* and *B*). A clear ring-like staining pattern for the IGF-1R was evident in both siITGB1 cell lines at 30 min, indicating that the IGF-1R was retained on the plasma membrane. Over time of adhesion, both Hs578T and HeLa siITGB1 cells began to spread and form focal cell adhesions, as indicated by vinculin and F-actin staining (Figs. 4, *A* and *B* and S4, *A* and *B*). Despite this, the IGF-1R remained at the plasma membrane in vinculin-containing focal adhesions, indicating that IGF-1R internalization is impaired in the absence of ITGB1 (Figs. 4, *A* and *B* and S4, *A* and *B*). At the latest time point (120 min), the IGF-1R was still retained on the plasma membrane in Hs578T siITGB1 cells, whereas in HeLa siITGB1 cells, the IGF-1R became internalized by 90 to 120 min (Figs. 4, *A* and *B* and S4, *A* and *B*).

Taken together, these data indicate that ITGB1 expression distinctly alters IGF-1R location in less migratory, hormone-responsive MCF-7 cells compared to more aggressive TNBC Hs578T breast cancer cells. Moreover, loss of ITGB1 protein expression in actively adhering migratory cancer cells promotes IGF-1R retention at the plasma membrane, impairing its internalization and intracellular trafficking.

### Loss of **β**_1_-integrin stabilizes WT and Tyr^1250/1251^ phosphomimetic IGF-1R protein levels and decreases IGF-1R signaling output in R- cells

We next sought to determine whether the role of ITGB1 in IGF-1R internalization and intracellular location is linked to adhesion-dependent phosphorylation of Tyr^1250/1251^, which promotes IGF-1R accumulation at the Golgi (32). To this end, we investigated how ITGB1 suppression affects the nonphosphorylatable FF IGF-1R mutant, compared to WT IGF-1R and the phosphomimetic EE IGF-1R mutant.

**Figure 5:**
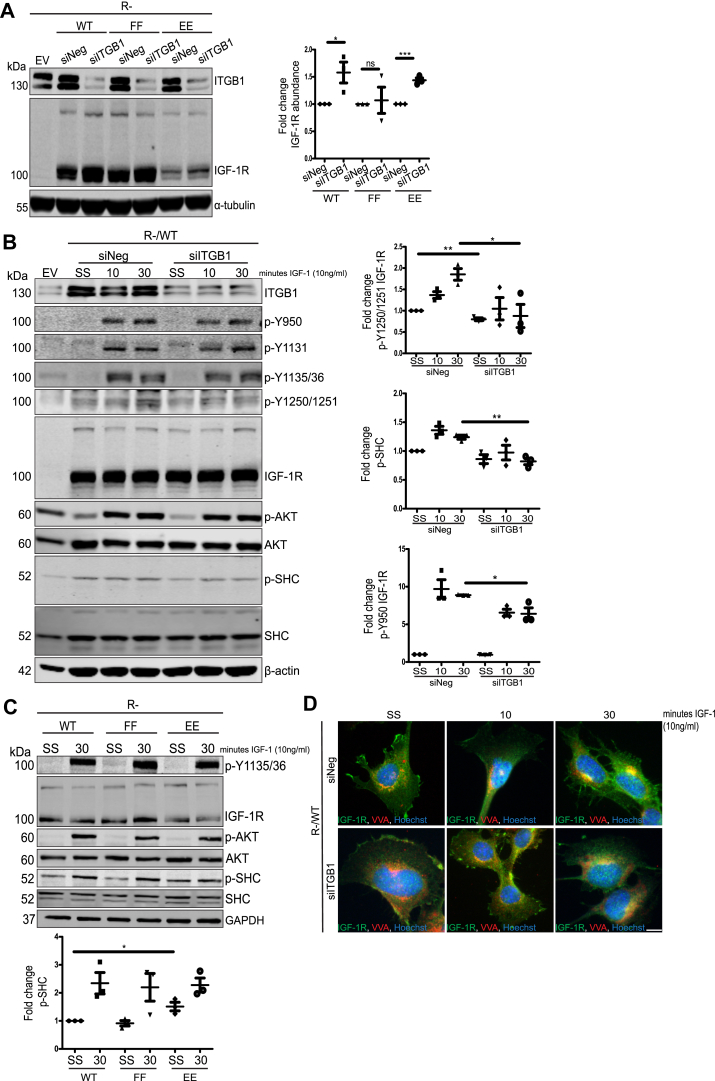
**ITGB1 suppression differentially affects IGF-1R turnover and signaling output in R- cells expressing WT, FF, or EE IGF-1Rs.***A*, R- cells transiently expressing either WT IGF-1R, FF mutant, or EE mutant, transfected with either siNeg or siITGB1 siRNA. Densitometry quantification is presented as the average fold change ± SEM in IGF-1R protein levels relative to the siNeg control sample (set to 1). *B*, R- cells transiently expressing IGF-1R WT, transfected with siNeg or siITGB1 siRNA. Cells were serum starved for 4 h and subsequently stimulated with 10 ng/ml IGF-1 for 10 min or 30 min. Densitometry quantification is expressed as the average fold change ± SEM in phosphoprotein levels normalized to total levels of the nonphosphorylated protein, which was normalized to loading control. All quantifications are graphed relative to the unstimulated control sample (set to 1). *C*, immunoblot of R- cells stably expressing WT IGF-1R, FF mutant, or EE mutant. Cells were serum starved for 4 h and subsequently stimulated with 10 ng/ml IGF-1 for 30 min. Densitometry quantification is expressed as the average fold change ± SEM in phosphoprotein levels normalized to total levels of the nonphosphorylated protein, which was normalized to loading control. Quantifications are graphed relative to the unstimulated control sample (set to 1) (*D*) immunofluorescence of R- cells transiently expressing WT IGF-1R, with or without ITGB1 suppression. Cells were serum starved for 4 h and then stimulated with 10 ng/ml IGF-1 for 10 min or 30 min. Cells were immediately fixed and then stained for IGF-1R in *green*, *cis*- and *trans*-Golgi (VVA) in *red*, and nuclei in *blue* (Hoechst nuclear stain). Magnification = 100×. The scale bar represents 20 μm. All experiments are representative of n = 3 independent experiments. Where relevant, significance was calculated using unpaired, two-tailed Student’s *t* test. Statistical significance was determined as *p*-value <0.05. Graded *p*-values are represented as follows: ∗*p* < 0.05, ∗∗*p* < 0.005, and ∗∗∗*p* < 0.0005. IGF-1R, insulin-like growth factor 1 receptor; ITGB1, β_1_-integrin.

First, we transiently expressed WT, FF, and EE IGF-1Rs in R- cells (mouse embryonic fibroblasts lacking endogenous IGF-1R (52)) and subsequently suppressed ITGB1 using siRNA. Suppression of ITGB1 significantly increased WT and EE IGF-1R protein abundance, but not that of the FF IGF-1R, relative to their respective siNeg controls (Fig. 5*A*). This indicates that turnover of ectopically expressed FF IGF-1R is independent of ITGB1 protein expression, in agreement with previous findings of this mutant’s inability to interact with the ITGB1-scaffolding protein, RACK1 (30).

Next, we evaluated how loss of ITGB1 protein expression affects IGF-1R signaling output in these cells. Again, we transiently expressed WT IGF-1R in R- cells and subsequently suppressed ITGB1. In response to IGF-1 stimulation, siITGB1 cells showed reduced IGF-1R autophosphorylation, specifically in the case of Tyr^950^ and Tyr^1250/1251^. This was accompanied by reduced phosphorylation of SHC (Fig. 5*B*). We also assessed whether mutation of the Tyr^1250/1251^ site to either FF or EE could influence IGF-1-induced SHC phosphorylation using R- cells stably expressing WT, FF, or EE IGF-1Rs. Here, we observed that for cells expressing WT and FF IGF-1Rs, SHC phosphorylation was reduced in serum starvation (SS) conditions and was subsequently increased in response to IGF-1 stimulation (Fig. 5*C*). However, in cells expressing EE IGF-1R, SHC exhibited higher basal phosphorylation in SS conditions (Fig. 5*C*) relative to cells expressing WT and FF IGF-1Rs. This suggests that mutation of Tyr^1250/1251^ to EE may promote unregulated signaling through IGF-1R.

Immunofluorescence was also used to determine whether loss of ITGB1 expression affects IGF-1R translocation to the Golgi in response to IGF-1 stimulation. Cells with ITGB1 suppression showed less Golgi-localized IGF-1R than siNeg control cells, in response to IGF-1 stimulation (Fig. 5*D*). From this, we conclude that ITGB1 is required to enhance IGF-1-induced IGF-1R adhesion-dependent signaling and trafficking from the plasma membrane to the Golgi apparatus.

Taken together, these data demonstrate that loss of ITGB1 expression increases IGF-1R protein abundance, only in the case of WT and phosphomimetic EE IGF-1Rs. Loss of ITGB1 also reduces IGF-1R autophosphorylation, particularly of Tyr^950^ and Tyr^1250/1251^, as well as IGF-1-induced phosphorylation of SHC. This coincides with delayed IGF-1R accumulation at the Golgi apparatus in response to IGF-1 stimulation. The putative phosphomimetic EE IGF-1R also promotes higher basal SHC phosphorylation in starvation conditions. Overall, these data suggest that phosphorylation of Tyr^1250/1251^, which promotes IGF-1R internalization, is required to integrate ITGB1 adhesion signals that influence IGF-1R protein turnover, activity, and subcellular location.

### EE phosphomimetic IGF-1R mutant supports a more transformed phenotype than WT IGF-1R or FF IGF-1R mutant

The results thus far suggest that ITGB1-mediated adhesion signaling promotes internalization and activity of the IGF-1R. This effect on IGF-1R may be mimicked or enhanced in cells expressing the EE IGF-1R mutant, which is rapidly internalized and degraded (32). While it has previously been demonstrated that R- cells expressing the FF IGF-1R mutant are impaired in cell migration, clonogenic growth, and cytoskeletal organization (30, 43, 53), the EE mutant, which mimics a constitutively phosphorylated Tyr^1250/1251^ site, has not yet been evaluated for these functions in cells (32). We hypothesized that a gain of function by this mutant would further support the role of phosphorylated Tyr^1250^^/^^1251^ in contributing to aggressive cancer cell phenotypes.

**Figure 6:**
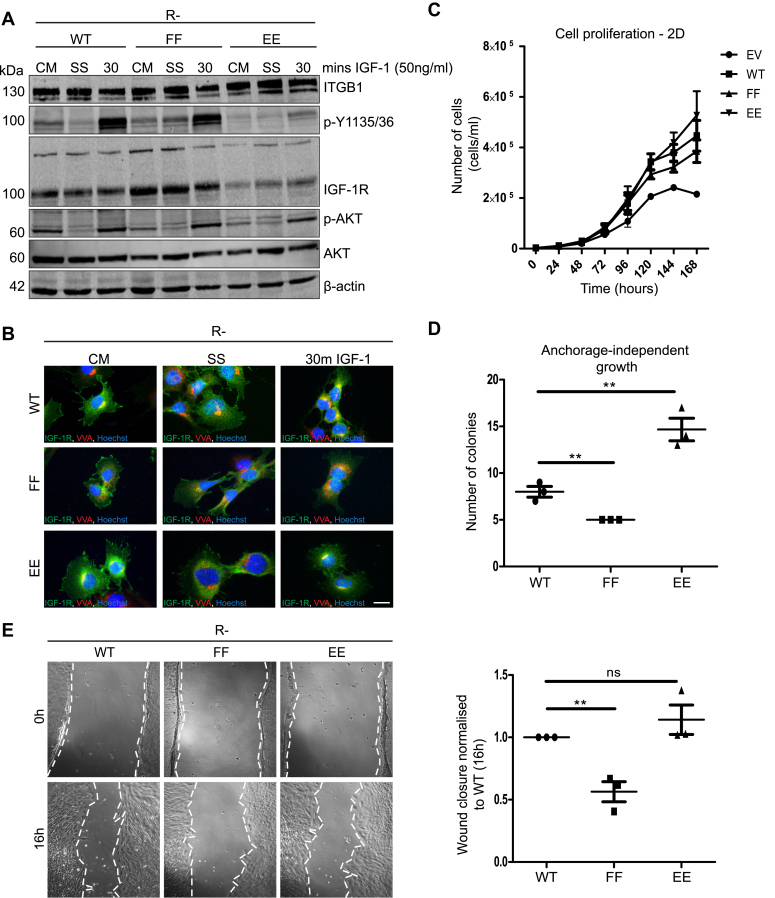
**R-cells stably expressing EE IGF-1R mutant exhibit a more transformed phenotype compared to WT or FF IGF-1R.***A*, immunoblot of R- cells stably expressing WT, FF, or EE IGF-1R. Cells were maintained in complete medium (CM), or serum starved (SS) for 4 h prior to stimulation with 50 ng/ml IGF-1 for 30 min. *B*, immunofluorescence images of R-cells stably expressing WT, FF, or EE IGF-1R (representative data for n = 3 experiments). Cells were stained for IGF-1R in *green*, the *cis*- and *trans*-Golgi (VVA) in *red*, with Hoechst staining nuclei in *blue*. Magnification = 100×. The scale bar represents 20 μm. *C*, proliferation assay of R- cells stably expressing EV (control), WT, FF, or EE IGF-1R. Cells were seeded at equal densities and then counted at each of the indicated time points. The data are representative of n = 3 independent experiments, each performed as technical triplicates. Error bars represent the SEM. *D*, anchorage-independent growth (soft agar colony formation) assay of R- cells stably expressing WT, FF, or EE IGF-1R. Cells were seeded at equal densities in soft agarose and cultured for 4 weeks. Colonies were stained with crystal violet and counted. Colonies with diameter >100 μm were counted under an inverted microscope. The data are representative of the mean colonies per well from n = 3 independent experiments, each performed as technical triplicates. Error bars represent the mean ± SEM. *E*, wound healing scratch (cell migration) assay of R- cells expressing WT, FF, or EE IGF-1R. Cells were seeded at equal densities and a wound was scratched in the confluent monolayer of each well. Images of wounds were captured immediately at 0 h and again at 16 h after wounding. Wound closure was quantified using ImageJ software. The data represent n = 3 independent experiments, performed as technical triplicates, and are graphed as the mean ± SEM. For (*D* and *E*), statistical significance was determined using an unpaired, two-tailed Student’s *t* test relative to WT sample, and was determined as *p*-value <0.05. Graded *p*-values are represented as follows: ∗*p* < 0.05, ∗∗*p* < 0.005, and ∗∗∗*p* < 0.0005. IGF-1R, insulin-like growth factor 1 receptor.

To test this, we generated a panel of R- cells that stably express WT and mutant receptors, selecting pools with WT and FF IGF-1Rs expressed at similar levels, and the EE IGF-1R expressed at slightly lower levels (Fig. 6*A*). The IGF-1R signaling output from these cells in response to IGF-1 stimulation was similar to our previous observations with transiently expressed mutants (Fig. 6*A*) (32). All three receptors were observed at the plasma membrane and at cell-matrix contact points in complete medium conditions, with the WT and EE receptors exhibiting more intracellular staining and Golgi colocalization than the FF receptor, which was retained on the plasma membrane (Fig. 6*B*). Following SS, the IGF-1R was excluded from the Golgi apparatus in all three cell lines; however, after 30 min IGF-1 stimulation, the WT and EE IGF-1Rs accumulated at the Golgi, while FF IGF-1R did not (Fig. 6*B*).

Cell proliferation in monolayer cultures did not differ significantly between R- cells expressing WT, FF, or EE mutants, with all three proliferating more over time than the control (empty vector) R- cells (Fig. 6*C*). However, the ability to form colonies in soft agarose was distinctly different, with R- cells expressing the EE IGF-1R mutant producing approximately 3-fold more colonies than R-/WT cells (Fig. 6*D*). Cells expressing the FF IGF-1R mutant produced fewer colonies than R-/WT cells, as previously demonstrated (42). In wound healing cell migration assays, R-/EE cells exhibited a similar capacity to R-/WT cells for wound closure, whereas cells expressing the FF IGF-1R mutant were impaired in cell migration, in agreement with previous studies (30, 38). These results demonstrate that mutation of the Tyr^1250/1251^ site to phosphomimetic EE residues confers a gain-of-function in IGF-1R-mediated phenotypes that support cellular transformation.

We conclude that phosphorylation of Tyr^1250/1251^ may enhance these IGF-1R functions in cancer cells, resulting in a more transformed phenotype. These data also support previous findings in which nonphosphorylatable FF IGF-1R fails to support cellular transformation (42). Taken together with our results thus far, these findings demonstrate a requirement for Tyr^1250/1251^ phosphorylation in integrating ITGB1-mediated adhesion signals with IGF-1R trafficking and functional output.

### Mutation of Tyr^1250/1251^ to phenylalanine or glutamic acid alters the conformation of the ^1248^SFYYS^1252^ motif

Our findings indicate that the protransformation/migration signals from the IGF-1R require phosphorylation of Tyr^1250/1251^, which reside in the CT. These tyrosines are not present in the IR, which has an almost identical kinase domain, but is most dissimilar in the amino acid sequence of its CT (45). The CT is implicated in distinguishing the protransformation, migration, and survival signaling potential of the IGF-1R compared to the IR (41, 46, 53).

**Figure 7:**
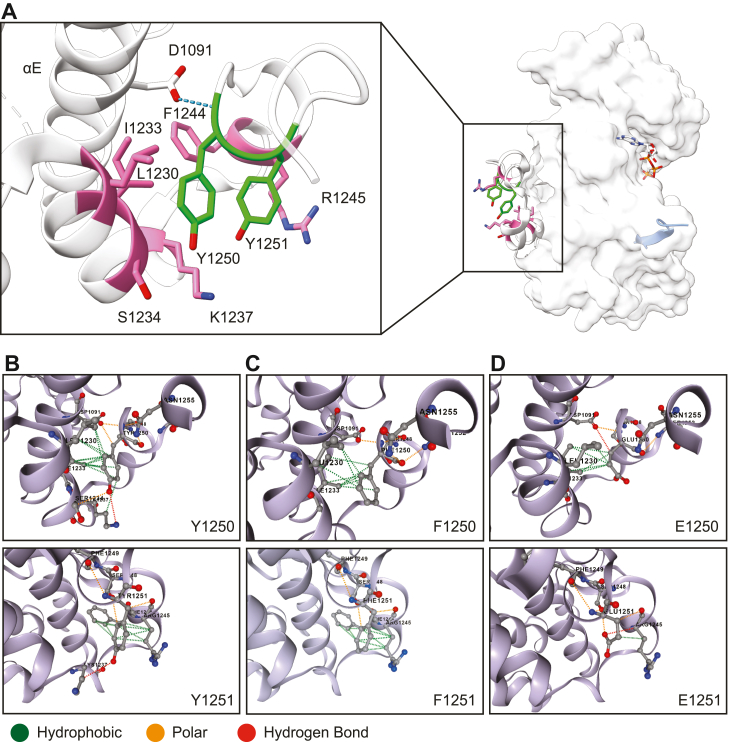
**Predicted residue interactions show changes in**^**1248**^**SFYYS**^**1252**^**motif conformation.***A*, structure of ligand-bound activated IGF-1R kinase (PDB 1K3A) bound to peptide substrate (*light blue*), with zooms showing the kinase-proximal region of the C-terminal tail, which contains the ^1248^SFYYS^1252^ motif. Y1250/Y1251 (*green*) and its contacting residues (*pink*) are denoted, as well as D1091 in helix αE, which is shown to hydrogen bond with the peptide backbone of Y1250. DynaMut2 predictions of IGF-1R WT (*B*) Y1250F/Y1251F (*C*) and Y1250E/Y1251E (*D*) show loss of polar contacts with K1237 in the case of the Y1250/1251 mutants. Specifically in the case of Y1250E/Y1250E, enhanced interaction with R1245 through hydrogen bonding was predicted, as well as loss of hydrophobic contacts with L1230, I1233, and F1244. These changes in residue contacts are expected to cause conformational changes that control positioning of distally located residues in the C-terminus. IGF-1R, insulin-like growth factor 1 receptor; PDB, Protein Data Bank.

Tyr^1250/1251^ forms part of the ^1248^SFYYS^1252^ motif, located in the kinase-proximal region of the CT. Although not part of the enzymatic kinase core, crystal structures show this portion of the CT packed tightly against the kinase C-lobe (Fig. 7*A*). Previously, we proposed that ^1248^SFYYS^1252^ functions as a conformational switch linking the kinase domain and CT, and that phosphorylation of Ser^1248^ within this motif induces a conformational change that allosterically controls CT positioning, and presentation of distally located residues (35). Here, we expanded this model to include tyrosine phosphorylation and to provide an explanation for the characteristics observed with the Tyr^1250/1251^ mutants.

To explore how phosphorylation of Tyr^1250/1251^ or mutation to phenylalanine or glutamic acid could alter the structural conformation of the ^1248^SFYYS^1252^ motif, we examined the interatomic interactions of Tyr^1250/1251^ in WT IGF-1R and subsequently modeled the local structural impact of the FF and EE mutations using the DynaMut2 tool (54). The structure of activated IGF-1R kinase (Protein Data Bank (PDB) 1K3A) (55) was used as the input for *in silico* predictions in DynaMut2. In WT IGF-1R, we observed that Y1250/Y1251 contacts were primarily confined to neighboring residues in the CT, although the peptide backbone of Y1250 does hydrogen bond with the sidechain of D1091 in helix αE (Fig. 7*A*), a residue that we previously proposed to be important for maintaining the position of the linker as seen in crystal structures (35). Y1250 establishes hydrophobic contacts with L1230 and I1233 through its aromatic ring, while the tyrosyl hydroxyl group is positioned to form polar contacts with S1234 and K1237. Y1251 establishes hydrophobic contacts with R1245 and F1244 through its aromatic ring, and the Y1251 hydroxyl group is positioned to form polar contacts with the sidechain of K1237 (Fig. 7*B*). It is conceivable that phosphorylation of these tyrosine residues could electrostatically stabilize interactions with K1237 (and R1245 in the case of Y1251).

For the FF mutant *in silico*, we observed that hydrophobic contacts were retained; however, tyrosyl polar contacts with S1234 and K1237 were lost, as was the potential for phosphorylation to strengthen interatomic interactions with K1237 and R1245 (Fig. 7*C*). We speculate that loss of the aforementioned polar contacts and the potential for phosphorylation could signify a loss of dynamics in this motif, which could hinder CT repositioning and presentation of distally located residues. This would be in accordance with experimental data where the FF mutant exhibits increased receptor stability, lower kinase autophosphorylation and activity, an inability to internalize, and deficiency in IGF-1R-dependent transformation phenotypes (cytoskeleton organization, migration, and anchorage independent growth) (30, 32, 38, 41, 42, 43, 56).

In the case of the EE mutant, although we observed the loss of tyrosyl hydroxyl group polar contacts with K1237, as we also saw with FF, the interaction between E1251 and R1245 was predicted to be enhanced by hydrogen bonding, potentially mimicking the enhanced interaction that would accompany phosphorylation of Y1251; however, it should also be acknowledged that loss of an aromatic ring at positions 1250 and 1251 may cause partial loss of hydrophobic contacts with L1230, I1233, and F1244 that could potentially contribute to a conformational change that may not occur for a phosphorylated tyrosine residue at these positions (Fig. 7*D*). Indeed, the DynaMut2 predicted Gibb’s free energy change (ΔΔG) (of unfolding) for EE was highly destabilizing (−2.68 kcal/mol), and only slightly destabilizing for FF (−0.68 kcal/mol), suggesting a profound conformational change in this local structure in the case of EE. This appears to be primarily due to the Y1250E substitution *in silico* as the predicted ΔΔG for Y1250E alone is −2.06 kcal/mol, while the predicted ΔΔG for Y1251E was slightly stabilizing (0.47 kcal/mol). However, our experimental data indicate that single E mutants at Y1250 and Y1251 recapitulate the effect of a double mutant in terms of receptor stability (Fig. S5). This would suggest that EE either (i) mimics the enhanced interaction of R1245 and residue 1251 that would occur with phosphorylated Y1251, or (ii) significantly alters the structural conformation in a manner that may not occur with phosphorylated tyrosine residues (*i.e.*, due to loss of hydrophobic contacts with surrounding residues). In the latter case, we speculate that a conformational change in EE may uncouple CT activity from the enzymatic core, resulting in an unregulated CT and constitutive presentation of distally located residues. This is in accordance with experimental observations with the EE mutant, which has similar kinase activity to WT but is rapidly internalized, exhibits decreased stability, and exhibits increased IGF-1R-dependent transformation phenotypes (Fig. 6).

We conclude that mutation of Tyr^1250/1251^ alters the conformation of the kinase-proximal ^1248^SFYYS^1252^ motif. In a physiological context, we propose that ITGB1 adhesion signals enhance phosphorylation of Tyr^1250/1251^, which may alter ^1248^SFYYS^1252^ conformation to control positioning/presentation of distally located CT residues. We speculate that this influences receptor signaling output in a manner exemplified by our observations with the FF and EE IGF-1R mutants.

## Discussion

The findings demonstrate that integrin-mediated adhesion signaling can control and enhance IGF-1R activity through the CT Tyr^1250/1251^ phospho-site, thereby promoting a transformed and migratory phenotype in aggressive cancers. The physiological and clinical relevance of adhesion-enhanced IGF-1R internalization and Golgi accumulation in cancer cell lines and fibroblasts are supported by the IGF-1R staining profile in TNBC tissue compared to nontumor breast tissue. In TNBC, loss of tissue architecture may contribute to IGF-1R dislocation or dispersal from the plasma membrane. This intracellular staining pattern is consistent with that observed in TNBC and other migratory cell lines, compared to hormone-responsive MCF-7 cells. Furthermore, the IGF-1R accumulates intracellularly at the Golgi in these aggressive cancer cell lines, and drug-induced disruption of the Golgi abrogates IGF-1-mediated activation of SHC (32). Thus, it is reasonable to conclude that intracellular IGF-1R signaling is a feature of aggressive cancers. This may contribute to the lack of efficacy of cell surface-targeted IGF-1R antibodies in cancers *versus* thyroid eye disease (11). Moreover, intracellularly located IGF-1R may serve as a putative biomarker for invasive tumors or those that may be unresponsive to IGF-1R targeting.

According to the TCGA PanCancer Atlas—Breast Invasive Carcinoma dataset, ITGB1 is strongly coexpressed with the IGF-1R in basal-like breast cancer patient tumor tissue. This is supported by high levels of ITGB1 expression in TNBC cell lines and is also evident in other migratory cell lines. Previous studies have implicated crosstalk between ITGB1 and IGF-1R in response to IGF-1 stimulation, particularly in breast cancer and prostate cancer cells (28, 30, 57). Our results indicate that ITGB1 is required to promote intracellular accumulation of the IGF-1R, by enhancing phosphorylation of Tyr^1250/1251^. We propose this as a dynamic cooperative process in turnover of IGF-1R-containing focal adhesion complexes, because ITGB1 is necessary for IGF-1R internalization in cells that are actively adhering to fibronectin. The requirement for IGF-1R in stabilizing ITGB1 (57) would suggest an opposing crosstalk with respect to IGF-1R *versus* ITGB1 protein turnover.

In addition, we show that ITGB1 signals are required to enhance autophosphorylation of IGF-1R on Tyr^950^ and Tyr^1250/1251^, which is in agreement with studies demonstrating that mutation of either Tyr^950^ or Tyr^1250/1251^ to nonphosphorylatable Phe impairs IGF-1R internalization (30, 32, 56, 58). This influences signaling from IGF-1R-containing cell adhesion complexes because activation of key signaling molecules, including SHC, is associated with internalized IGF-1R (58). This conclusion is supported by our observation that ITGB1 signals are required for IGF-1-induced phosphorylation of SHC, which is consistent with studies showing that mutation of Tyr^950^ to nonphosphorylatable Phe impairs SHC recruitment and activation (59). Furthermore, overexpression of RACK1, which complexes ITGB1 and IGF-1R to key signaling molecules that coordinate cell migration (*e.g.*, SHC, Shp-2, IRS-2, FAK, and Src (24, 27, 30)), skews IGF-1R signaling away from AKT toward SHC (60). Taken together with the findings here, this would suggest that the ITGB1-IGF-1R complex is required for IGF-1R internalization and SHC activation during focal adhesion turnover.

Our data indicate that a phosphorylated Tyr^1250/1251^ in the IGF-1R is necessary to integrate ITGB1 and IGF-1R signaling. This builds upon our and others’ findings that mutation of these tyrosines to phenylalanine impairs recruitment of IGF-1R into a complex with ITGB1, and results in a receptor with impaired internalization and activity, as well as disrupted IGF-1 stimulated migration, cytoskeletal organization, and clonogenic growth (30, 32, 38, 41, 42, 43, 44, 56). The stability of this IGF-1R mutant is not significantly affected by ITGB1 suppression, although, ITGB1 suppression significantly increases the levels of WT and EE IGF-1Rs. This indicates that a phosphorylated Tyr^1250/1251^ site is required for IGF-1R to interface with ITGB1 signals that promote IGF-1R internalization and turnover. Our conclusions are supported by observations with the EE mutant, which is rapidly internalized and turned over (32). This is associated with enhanced cell migration and anchorage-independent growth. In effect, the EE mutant imitates the integrin cooperative signal to promote a transformed cellular phenotype.

In different cellular contexts, it is possible that integrin ligation and signaling enhance IGF-1R activity, with integrin acting as an intermediary for other receptors and kinases. For example, the tissue factor and coagulation factor VII (TF-fVIIa) complex has been shown to engage with ITGB1 to promote IGF-1R nuclear translocation and cell survival in a process involving caveolin modification (61). Furthermore, integrin-activated Src family tyrosine kinases (SFKs) are implicated in crosstalk between IGF-1R and integrins (62). In addition, the SFK substrate, p130Cas/BCAR1, which is regulated by IGF-1R signaling (63, 64), is known to coordinate ITGB1-stimulated focal adhesion assembly (65). Another SFK family member, Yes, is implicated in resistance to IGF-1R inhibitors in rhabdomyosarcoma (66). Integrin activity may also be enhanced by protease-activated receptors (PARs) and G protein-coupled receptors (GPCRs) (67), which could, in turn, enhance IGF-1R internalization and activity.

The IGF-1R CT, which contains Tyr^1250/1251^, distinguishes IGF-1R and IR signaling, and enhances IGF-1R signaling that supports cellular transformation and aggressive cancer phenotypes (30, 32, 38, 40, 41, 42, 43). We previously proposed that phosphorylation of Ser^1248^, positioned adjacent to Tyr^1250/1251^ in the kinase-proximal ^1248^SFYYS^1252^ motif, controls trajectory of the distal CT relative to the kinase domain, promoting an autoinhibitory conformation (35). We hypothesize that Tyr^1250/1251^ phosphorylation may also alter ^1248^SFYYS^1252^ conformation to control positioning of the distal CT but in an opposing manner by preventing the distal CT from adopting an autoinhibitory position, and that this phosphorylation event requires an ITGB1 adhesion context.

To explain why the activities of FF and EE IGF-1Rs differ from WT, we assessed the interatomic residue interactions of Tyr^1250/1251^ and predicted how the local structural impact of FF and EE substitutions may alter positioning of the distal CT in a manner that integrates with the phenotypes observed. Without these tyrosine residues, the FF and EE IGF-1R mutants appear to no longer conform to cellular context, and, thus, may not fully recapitulate WT IGF-1R CT dynamics, instead representing static snapshots of IGF-1R CT behavior. In the FF IGF-1R mutant, we predict that loss of phosphorylation may hinder CT dynamics, including presentation of distally located residues that control autophosphorylation, kinase activity, and receptor internalization. We predict that the EE IGF-1R mutant acts in an opposing manner, either as a phosphomimetic or because of an altered structural conformation driven by loss of aromatic residues. This may uncouple CT activity from the enzymatic core, resulting in an unregulated CT and constitutive presentation of distally located residues that drive receptor internalization, turnover, and activity.

It is possible that phosphorylation of Tyr^1250/1251^ could prevent phosphorylation of Ser^1248^ by locking the ^1248^SFYYS^1252^ motif in an “active” conformation that prevents the distal CT from adopting an autoinhibitory position. In this manner, Ser and Tyr phosphorylation of the ^1248^SFYYS^1252^ motif may function in distinct ways to regulate IGF-1R CT behavior. Although conformational changes elicited by phosphorylation/mutation may alter the positioning of distally located residues in the CT, specific motifs that potentiate the cellular effects of FF and EE IGF-1Rs remain unclear.

In summary, our findings demonstrate that cell context-dependent ITGB1 adhesion signals dictate IGF-1R subcellular localization and are required for IGF-1R internalization, Tyr^1250/1251^ phosphorylation, and receptor signaling outputs that support migratory and aggressive cancer phenotypes. This distinguishes IGF-1R and IR CTs and how they regulate receptor signaling outputs. We propose that intracellular IGF-1R may serve as a putative biomarker for aggressive cancers, and that therapeutic approaches targeting both ITGB1 and IGF-1R may increase IGF-1R cell surface presence to enhance therapeutic efficacy of cell surface-targeted anti-IGF-1R monoclonal antibodies.

## Experimental procedures

### Cell culture and IGF-1 stimulation

All cell lines were maintained in Dulbecco’s modified Eagle medium (DMEM)—medium-high glucose (Sigma-Aldrich #D6429), supplemented with 10% (v/v) heat inactivated-fetal bovine serum (FBS) (Thermo Fisher Scientific #10270106), 2 mM L-glutamine (Merck Life Science Ltd #G7513), and containing 100U/ml penicillin and 100 μg/ml streptomycin (Merck Life Science Ltd #P4333) at 37 °C, 5% CO_2_ in a humidified incubator.

For IGF-1 stimulation, cells were seeded 24 h before and allowed to reach approximately 70% confluence, washed three times with PBS, and then maintained in serum-free medium for 4 h, before addition of 10 ng/ml or 50 ng/ml IGF-1 (PeproTech #100-11) for the indicated times. All cell lines were originally purchased from American Type Culture Collection and authentication established by PCR-single-locus-technology (Eurofins Medigenomix, Forensik GmbH). Cells were determined to be free of *mycoplasma* by specific DNA staining and routinely maintained in culture for up to 6 to 8 weeks for use in experiments.

### siRNA and plasmid transfection

For siRNA knockdown experiments, cells were cultured for 24 h and allowed to reach 90% confluence, before removal using Trypsin-EDTA solution (Merck Life Science Ltd #T4174) and suspension in antibiotic-free medium. Oligonucleotide siRNAs (sequences in Table S1) were diluted in Opti-MEM (Thermo Fisher Scientific #51985026) to give a final concentration of 20 nM. The Lipofectamine RNAiMAX transfection reagent (Thermo Fisher Scientific #10514953) was prepared separately, as per manufacturer’s specifications, in Opti-MEM medium and incubated at room temperature (RT) for 5 min. The transfection reagent solution was added to the siRNA solution at a 1:1 ratio and left for 10 min at RT, before addition to the cell culture plates. Cells were subsequently seeded into the transfection solution and placed at 37 °C, 5% CO_2_ in a humidified incubator. Cells were reseeded for experiments 24 h later. Optimal knockdown efficiency was achieved 48 to 72 h posttransfection.

Hs578T cells were transfected with the GalT-EGFP plasmid (Addgene plasmid #11929 from Jennifer Lippincott-Schwartz lab) using Lipofectamine 2000 Transfection Reagent (Thermo Fisher Scientific #10696153), whereas MCF-7 cells were transfected with Lipofectamine (Thermo Fisher Scientific #18324-012). Cells were seeded 24 h prior to transfection in antibiotic-free medium. The transfection reagent and plasmid DNA (2 μg/6-well plate, 4 μg/6 cm plate, or 8 μg/10 cm plate) were diluted, separately, in Opti-MEM and left to incubate at RT for 5 min. Following this, the transfection reagent solution was mixed with the plasmid DNA solution at a 1:1 ratio and left for 25 min at RT before adding to cells that were then left to incubate at 37 °C, 5% CO_2_ for 4 to 6 h. After this, the transfection reagent was removed and replaced with fresh complete medium. For MCF-7 cells transfected with Lipofectamine, the transfection reagent was replaced with antibiotic-free medium containing 20% FBS. Cells were reseeded for experiments 24 h after transfection.

### Lentiviral transduction of R-cells for stable expression or WT IGF-1R or mutants

Complementary DNAs encoding WT IGF-1R, YY1250/1251FF, and YY1250/1251EE (32) were subcloned into the N174-MCS (Puro) vector (from Adam Karpf, Addgene plasmid #81068) using restriction enzyme digestion. Lentiviral particles containing the N174-MCS (Puro)-IGF-1R constructs were generated in HEK293T cells that were cultured at a density of 1.2 × 10^6^ cells per 10 cm plate, in 10 ml of antibiotic-free DMEM plus 10% FBS for 24 h prior to transfection. Each N174-MCS/IGF-1R transfer plasmid (2 μg) was combined with 1.5 μg psPAX2 packaging plasmid, and 500 ng pMD2.G envelope plasmid in a final volume of 40 μl serum-free Opti-MEM and 15 μl of FuGENE HD transfection reagent (Promega #E2311). Following incubation at RT for 30 min each DNA:FuGENE mix was added dropwise to a 10 cm plate of HEK293T cells. After 15 h, the transfection reagent was removed and replaced with 10 ml fresh DMEM + 10% FBS + penicillin/streptomycin. Viral particles were harvested, filtered, and stored at −80 °C at 48 h and 72 h post transfection.

R- cells were cultured to be 70% confluent at the time of infection. Prior to infection, the medium was replaced with 7 ml fresh culture media containing 6 μg/ml polybrene, followed by 3 ml of the required lentiviral particle solution. After 48 h, virus-containing medium was replaced with fresh medium containing 1.5 μg/ml puromycin to select for transduced R- cells. An uninfected plate of cells was maintained in parallel to serve as a positive control for puromycin selection. Following 48 h of selection, cells were passaged as normal. Expression of IGF-1R WT, FF, and EE was assessed by western blotting and immunofluorescence.

### Immunofluorescence with tissue sections and cultured cells

Slides mounted with fresh frozen tissue sections from TNBC tumor and contralateral, nontumor tissues were obtained from the Breast Cancer Now Tissue Bank (Barts Cancer Institute, Queen Mary University of London, London, UK). Slides were removed from storage at −80 °C and left at RT for 10 min. Sections were fixed by incubation in freshly made 4% paraformaldehyde (Thermo Fisher Scientific #11400580) diluted in PBS for 15 min at RT, then washed with PBS before being permeabilized and blocked by incubation in PBS/5% donkey serum (Sigma-Aldrich #D9663)/0.1% Triton X-100 (Sigma-Aldrich #T9284) for 30 min at RT. Primary antibodies at the indicated dilutions were prepared in PBS/5% donkey serum/0.05% Triton X-100, and incubated overnight (O/N) with tissue sections at 4 °C. Tissues were washed three times with PBS before being incubated with secondary antibodies and Hoechst nuclear stain (1:750 dilution) at RT for 60 min. Tissues were again washed three times with PBS before adding coverslips. Primary antibodies were as follows: anti-IGF-1R, CST #3027 (1:100); anti-GM130, BD catalog #610822 (1:400). Secondary antibodies were as follows: AlexaFluor 488 anti-rabbit, Jackson ImmunoResearch #711-545 to 152 (1:200); Cy3 anti-mouse, Jackson ImmunoResearch #715-165 to 150 (1:500). Images were captured using a Leica DMLB upright fluorescence microscope fitted with a Nikon DS-Fi1c camera and Nikon Image Studio Elements AR software (https://www.microscope.healthcare.nikon.com/products/software/nis-elements/nis-elements-advanced-research).

For immunofluorescence, cells were seeded on glass coverslips in cell culture plates and allowed to adhere O/N. At experimental end points, coverslips were removed from cell culture plates and washed gently with PBS. Cells were fixed by incubation in PBS containing 4% paraformaldehyde for 30 min at RT, washed three times with PBS, followed by quenching in 50 mM ammonium chloride solution for 20 min and further washing with PBS for 5 min. Cells were then permeabilized with PBS/0.1% Triton-X 100 for 5 min, washed three times with PBS and blocked by incubation in PBS containing 5% donkey serum for 60 min at RT. Coverslips were placed on 35 μl drops of the indicated primary antibodies, prepared in PBS/5% donkey serum, and incubated in a humidified chamber O/N at 4 °C. The following primary antibodies were used: anti-IGF-1Rα chain, α-IR-3, Calbiochem/Merck #GR11 (1:200); anti-IGF-1Rβ in human cells, CST #3027 (1:200), in mouse cells, CST #9750 (1:200); the *cis*-Golgi apparatus in human cells, GM130 BD catalog #610822 (1:200); the *cis*- and *trans*-Golgi compartments in mouse cells, biotinylated VVA Lectin Vector Laboratories #B-1235-2 (1:400); anti-vinculin, Sigma-Aldrich #V9131 (1:200); anti-β_1_-integrin inactive form, mAb 13, Sigma-Aldrich #MABT821 (1:300); anti-β_1_-integrin active form, mAb 2247, Sigma-Aldrich #MAB2247 (1:200). Coverslips were washed three times with PBS and then incubated, in the dark, for 60 min at RT in 35 μl drops of the appropriate secondary antibodies (AlexaFluor 488 anti-mouse, Jackson ImmunoResearch #715–545–150 (1:200); AlexaFluor 488 anti-rabbit, Jackson ImmunoResearch #711-545-152 (1:200); Cy3 anti-mouse, Jackson ImmunoResearch #715-165-150 (1:1000); Cy3 anti-rabbit, Jackson ImmunoResearch #711-165-152 (1:1000). Hoechst was used to stain nuclei (1:1000), and tetramethyl rhodamine isothiocyanate phalloidin, Sigma-Aldrich #P1951 or AlexaFluor 405 phalloidin, Thermo Fisher Scientific #A30104, was used to stain F-actin (1:1000)). After secondary antibody incubation, coverslips were washed three times with PBS and then mounted on glass slides using warm vinol mounting medium (16.5% Celvol #V-205S, 33% absolute glycerol and 0.1% sodium azide in PBS pH 8.5). Slides were washed with deionized water and dried O/N at RT before viewing using a Leica DMLB upright fluorescent microscope. Images were captured using a Nikon DS-Fi1c camera and Nikon Image Studio Elements AR software and processed using ImageJ software (https://imagej.net/ij/).

### Cell lysis, SDS-PAGE, and western blotting

At experiment endpoints, cell culture medium was removed from the culture plates and cells were washed once with ice-cold PBS. The desired volume of RIPA lysis buffer (50 mM Tris pH 8.0, 150 mM sodium chloride, 1% NP-40, 0.5% sodium deoxycholate, 0.1% SDS, made up in deionized water), freshly supplemented with 1/100 Pierce HALT Protease Inhibitor Cocktail (Thermo Fisher Scientific #78439), 2 mM sodium orthovanadate (Sigma-Aldrich #S6508), 2.5 mM sodium pyrophosphate (Sigma-Aldrich #221368), and 1 mM β-glycerophosphate (Merck Life Science Ltd #35675) was then added to each plate. The cells were then scraped into the lysis buffer using a cell scraper, and the lysis buffer-cell solution was harvested in a 1.5 ml microfuge tube. Samples were subsequently incubated on ice for 30 min and then centrifuged for 15 min at 20,000*g* in a precooled benchtop centrifuge. Protein concentrations were determined by Bradford assay (Sigma-Aldrich #B6916).

Samples (containing 20–60 μg protein) were boiled in SDS-PAGE loading buffer at 95 °C for 5 min and then loaded to gels. Separated proteins were then transferred to nitrocellulose membranes using a western blot wet transfer apparatus (Bio-Rad #1703930) in wet transfer buffer (25 mM Tris and 192 mM glycine in deionized water) at a constant current of 250 mA for 70 min. Membranes were blocked with Tris-buffered saline (TBS) containing 0.1% Tween-20 (TBS-T) and 5% bovine serum albumin (BSA) (Sigma-Aldrich #A2153) for 60 min at RT and incubated with primary antibody O/N at 4 °C. All primary antibodies (listed in Table S2) were prepared at 1:1000 dilution in TBS-T/5% BSA and 1:600 sodium azide (Sigma-Aldrich S2002). Primary antibodies were removed, and membranes were washed three times with TBS-T, and then incubated with the appropriate IRDye secondary antibodies diluted 1:10,000 in 5% nonfat milk in TBS-T (LI-COR, IRDye 680 anti-mouse #926–68070, IRDye 680 anti-rabbit #926–68071, IRDye 800 anti-mouse #926–32210, and IRDye 800 anti-rabbit #926–32211) for 60 min at RT. Membranes were then washed twice with TBS-T, and once with TBS. Finally, western blots were scanned using a LI-COR Odyssey Infrared Imager and analyzed using LI-COR Image Studio software (https://www.licor.com/bio/image-studio/). Densitometry measurements were performed using LI-COR Image Studio Lite software.

### Cell adhesion assays

Cell culture plates were coated with 5 μg/ml fibronectin (Sigma-Aldrich #F1141) diluted in PBS and left O/N at 4 °C. Coated plates were washed three times with PBS and blocked with 2.5% BSA diluted in PBS for 60 min at 37 °C. After blocking, the plates were washed three times with PBS and 6 × 10^5^ cells/well were seeded onto the fibronectin-coated plates for the indicated time points, before fixation for immunofluorescence or harvesting for cell lysis.

### Cell migration assays

R- cells stably expressing WT, FF, or EE IGF-1R were cultured in 24-well plates at 4 × 10^5^ cells/ml (500 μl per well) for 24 h, at which time a wound was scratched in the confluent monolayer using a sterile p20 pipette tip. Cells were photographed at 0 h (immediately after wounding) and again 16 h later. Cells were observed using a Nikon Eclipse TE300 inverted microscope. All wounds were photographed at 10× magnification and images captured using a Hamamatsu C11440 Digital Camera with Nikon Image Suite Elements AR software. ImageJ software was used to quantify wound closure efficiency.

### Cell proliferation assays

R- cells stably expressing WT, FF, or EE IGF-1Rs were seeded in triplicate in 24-well plates at a density of 3 × 10^3^ cells/well. After 24, 48, 72, 96, 120, 144, or 168 h, the cells were washed, trypsinized, and counted. The average cell number for each sample at each time point was calculated and the proliferation rates determined by plotting cell number *versus* each time point.

### Soft agarose clonogenic assays

Low-melting point agarose (Sigma-Aldrich #A9414) was added to each well of a 6-well plate (2 ml of 0.8% agarose) and left to solidify. Cell suspensions were prepared in 7 ml complete medium and then diluted 1:1 in 7 ml of 0.8% warm agarose solution (resulting in 14 ml 0.4% cell/agarose/complete medium solution). Subsequently, 2 ml of this cell/agarose/medium solution (3 × 10^4^ cells) was then added to each well containing the solidified 0.8% agarose and left to solidify. Following this, 2 ml of complete medium was added to each well. Cells were then placed in a humidified incubator at 37 °C, 5% CO_2_ and left for 4 weeks to allow colonies to form. Media were replaced every 3 to 4 days. After 4 weeks, media were removed and 2 ml of freshly filtered 0.05% crystal violet dissolved in 20% ethanol was added to each well and left to stain at 4 °C O/N. The plates were subsequently destained by repeated washing with deionized water, and the number of colonies >100 μm in diameter was quantified by examination at 10× magnification using a Nikon Eclipse TE300 inverted microscope.

### IGF-1R structure modeling

Molecular visualization and annotation of the tris-phosphorylated IGF-1R kinase (PDB 1K3A) (55) was performed using UCSF ChimeraX, developed by the Resource for Biocomputing, Visualization, and Informatics at the University of California, San Francisco, with support from National Institutes of Health R01-GM129325 and the Office of Cyber Infrastructure and Computational Biology, National Institute of Allergy and Infectious Diseases (68). To investigate the effect of mutations on interatomic interactions, PDB 1K3A was used as the structural input for the DynaMut2 (https://biosig.lab.uq.edu.au/dynamut2/) server and single-point mutations were made as described. To estimate Gibbs free energy change (ΔΔG), double mutants were submitted to the server as multiple mutations. ΔΔG values less than 0 indicate destabilization, whereas ΔΔG values greater than 0 indicate stabilization (54).

### Bioinformatics and statistical analysis

The results shown in Figure 2, *A* and *B* are, in whole, based upon data generated by TCGA Research Network, https://www.cancer.gov/tcga. Protein expression data were downloaded from the TCGA PanCancer Atlas—Breast Invasive Carcinoma dataset (49), *via* cBioPortal (48). The IGF-1R, ITGB1, and ITGB3 protein expression data from this dataset that were used in this study were quantified by mass spectrometry by The National Cancer Institute’s Clinical Proteomic Tumor Analysis Consortium.

All statistical analyses were performed using GraphPad Prism Software (https://www.graphpad.com/). For data comparing the fold-change of samples in a group relative to one-another, statistical significance was determined using an unpaired, two-tailed, Student’s *t* test. Statistical significance was determined as *p*-value <0.05. Graded *p*-values are represented as follows: ∗*p* < 0.05, ∗∗*p* < 0.005, and ∗∗∗*p* < 0.0005.

## Data availability

All data from this study are included in the manuscript and supporting information.

## Supporting information

This article contains supporting information.

## Conflict of interest

The authors declare that they have no conflicts of interest with the contents of this article.
