# Supplementary material for: β1-integrin controls IGF-1R internalization and intracellular signaling
Source: J Biol Chem. 2024 Nov 27;301(1):108021. doi: 10.1016/j.jbc.2024.108021 (PMC11732470; doi:10.1016/j.jbc.2024.108021)
Supplement: Supporting Information [file mmc1.pdf]

S-1 – The IGF-1R is dispersed from the plasma membrane and throughout the cytoplasm in TNBC (**in reference to Fig. 1**).

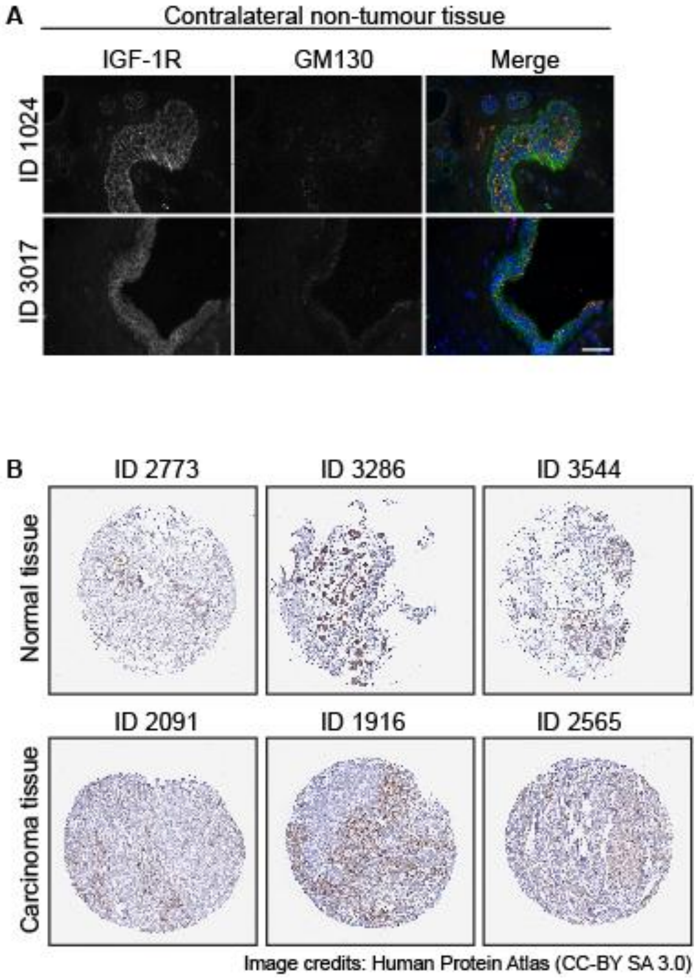

Figure S1 – The IGF-1R is dispersed from the plasma membrane and throughout the cytoplasm in TNBC.

(**A**) Immunofluorescence staining of contralateral, non-tumour tissue from TNBC patients. IGF-1R in green, the Golgi marker GM130 is in red, and nuclei are stained blue with Hoechst stain. Magnification = 40X. Scalebar = 200µm. (**B**) DAB (3,3'-diaminobenzidine) staining of IGF-1R in normal breast tissue (top panels) and breast carcinoma tissue (bottom panels). Image credit: Human Protein Atlas (CC-BY SA 3.0). Images available from v14.proteinatlas.org.

S-2 – ITGB1 is highly expressed in migratory cell lines (**in reference to Fig. 2**).

A

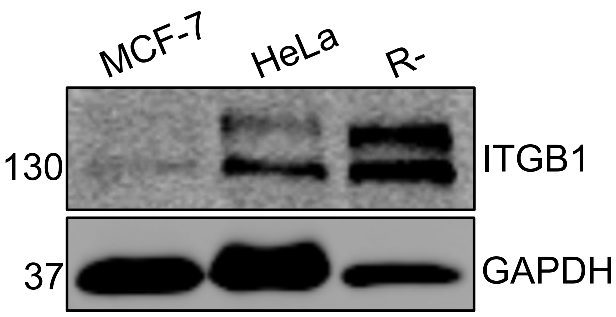

Figure S2 – ITGB1 is highly expressed in migratory cell lines.

(**A**) Immunoblot showing the expression of ITGB1 in MCF-7 (low migratory control), HeLa, and R- cell lines.

S-3 – IGF-1R does not co-accumulate with ITGB1 at the Golgi apparatus (**in reference to Fig .3**).

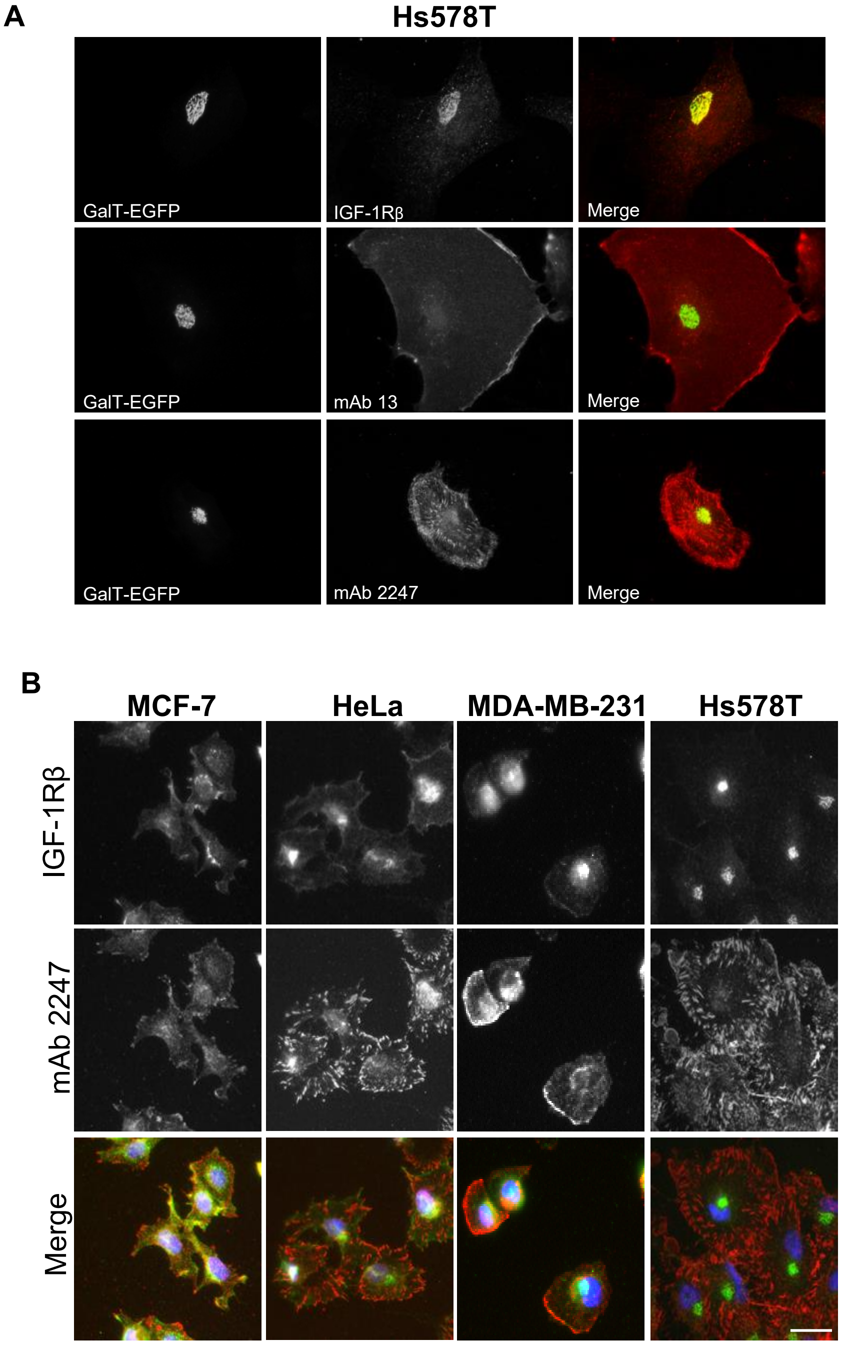

Figure S3 – IGF-1R does not co-accumulate with ITGB1 at the Golgi apparatus.

(**A**) Immunofluorescence staining of Hs578T cells transfected with an EGFP-tagged Golgi-localised construct, GalT-EGFP. Cells were stained for either IGF-1R, inactive ITGB1 (mAb 13), or active ITGB1 (mAb 2247) – all shown in red. Magnification = 100X. Scale bar = 20µm. (**B**) Immunofluorescence staining of MCF-7, HeLa, MDA-MB-231, and Hs578T cancer cells. IGF-1Rβ chain is shown in green, active ITGB1 (mAb 2247) is shown in red, and nuclei are stained blue with Hoechst stain. Magnification = 40X. Scalebar = 100µm. All experiments representative of n = 3 independent experiments.

S-4 – IGF-1R is retained on the plasma membrane upon adhesion to fibronectin in cells with ITGB1 suppression (**in reference to Fig. 4**).

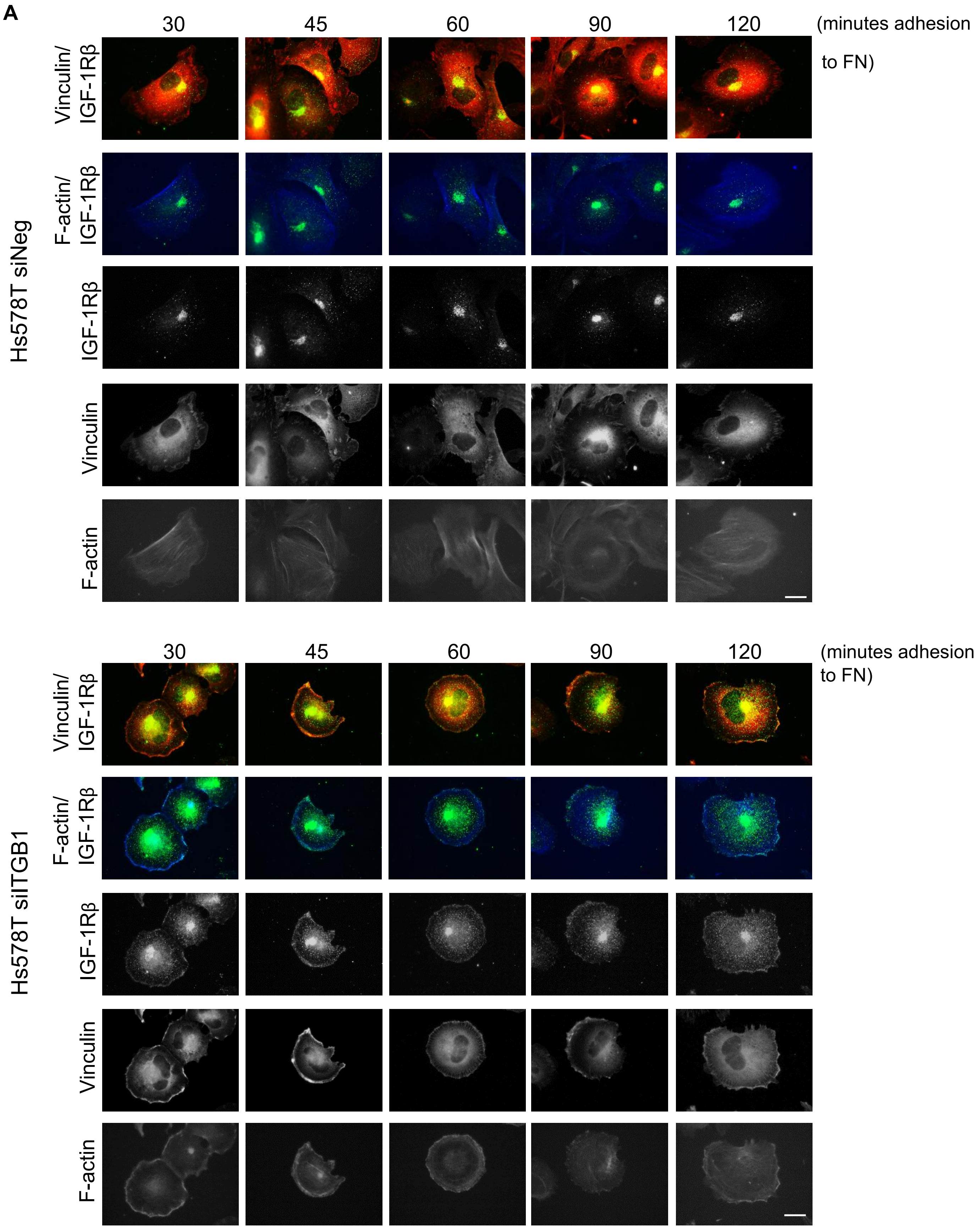

S-4 (continued)

B

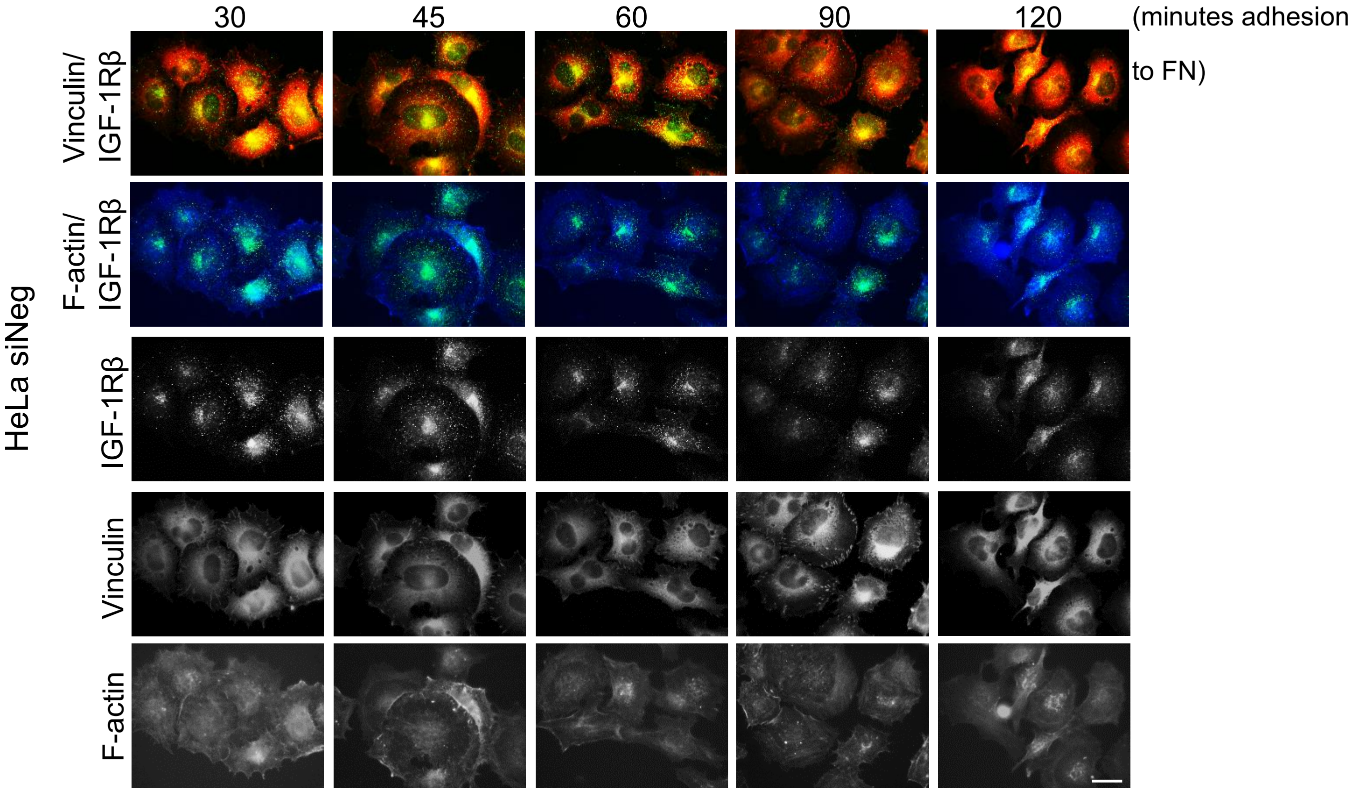

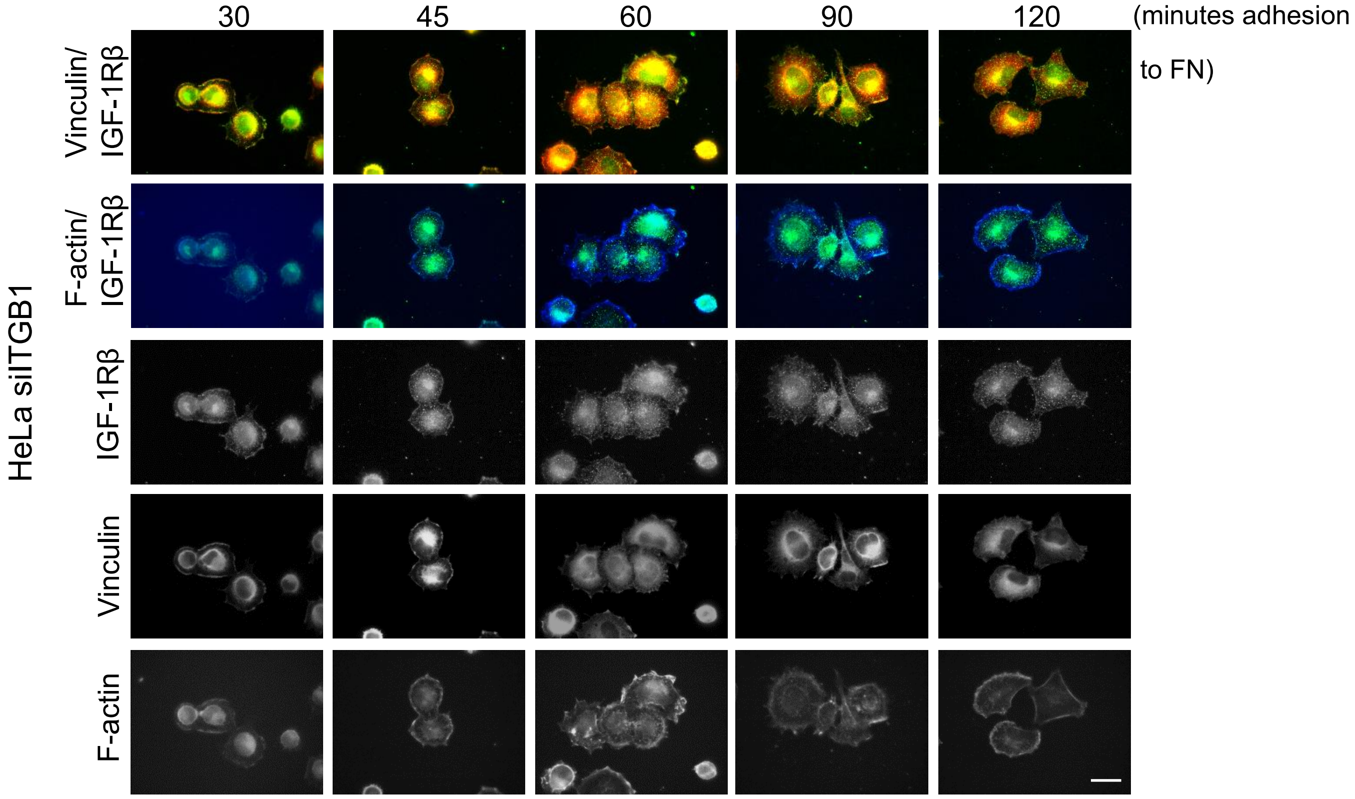

Figure S4 – IGF-1R is retained on the plasma membrane upon adhesion to fibronectin in cells with ITGB1 suppression.

(**A**) Hs578T cells and (**B**) HeLa cells transfected with siNeg or siITGB1. Cells were seeded 48 hrs after transfection on fibronectin-coated coverslips and left to adhere for the indicated timepoints prior to fixation for immunofluorescence. Cells were stained for IGF-1Rβ chain (green), vinculin (red), and F-actin (blue). For all images, magnification = 100X, scalebars = 20μm. Representative of n = 3 independent experiments.

S-5 – IGF-1R Y1250E and Y1251E single mutants exhibit similar stability to Y1250E/Y1251E double mutant in MCF-7 IGF-1R knockout cells (**in relation to Fig. 7**).

A

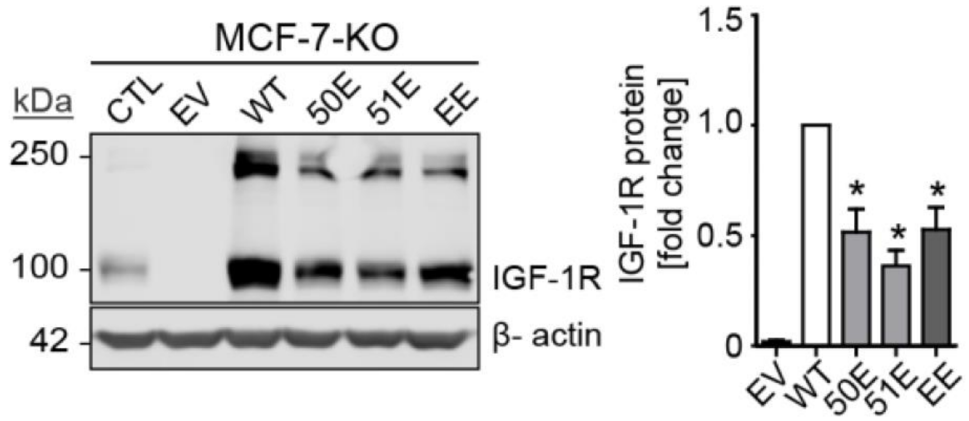

Figure S5 – IGF-1R Y1250E and Y1251E single mutants exhibit similar stability to Y1250E/Y1251E double mutant in MCF-7 IGF-1R knockout cells.

(**A**) Western blot analysis of MCF-7 IGF-1R knockout cells transiently transfected with empty vector (EV) plasmid, IGF-1R WT, IGF-1R Y1250E mutant (50E), IGF-1R Y1251E mutant (51E), or IGF-1R Y1250/1251E double mutant (EE). Lysate from parental MCF-7 cells included as control (CTL). Densitometry quantification is presented as the average fold change ± SEM in IGF-1R protein levels (normalised to loading control) relative to the WT IGF-1R (set to 1), indicated as a white bar on all graphs. Representative of n = 3 independent experiments. Significance was calculated using unpaired, two-tailed Student's *t*-test. Statistical significance was determined as *P*-value <0.05. Graded *P*-values are represented as follows: * *P*<0.05, ** *P*<0.005, and *** *P*<0.0005.

Supporting Methods

HPA IGF-1R DAB tissue staining.

IGF-1R 3,3'-diaminobenzidine (DAB) breast tissue staining images in Fig. S1 (B) were produced by, and obtained from, the Human Protein Atlas (HPA) program: proteinatlas.org (65). Images available at the following links: v14.proteinatlas.org/ENSG00000140443-IGF1R/tissue/breast and v14.proteinatlas.org/ENSG00000140443-IGF1R/cancer/tissue/breast+cancer.

Tables

Table S1 – siRNA sequences

**siRNA**	**Sequence (5' → 3')**	**Final concentration**
siNeg	CGUUAAUCGCGUAUAAUACGCGUA	20nM
siITGB1	siRNA 1: GUGCAGAGCCUUCAAUAAA<br>siRNA 2: GGUAGAAAGUCGGGACAAA<br>siRNA 3: UGAUAGAUCCAAUGGCUUA<br>siRNA 4: GGGCAAACGUGUGAGAUGU	20nM

Table S2 – Antibodies used for western blotting

**Antibody Target Protein**	**Company**	**Catalogue Number**
β1-integrin	Abcam	ab183666
IGF-1Rβ	CST	3027
p-Y950 IGF-1R	CST	4568
p-Y1131 IGF-1R	CST	3021
p-Y1135/1136 IGF-1R	CST	3024
p-Y1250/1251 IGF-1R	Santa Cruz	sc-293102
AKT	CST	2920
p-AKT (Ser473)	CST	4060
SHC	BD Transduction Labs	610879
p-SHC (Tyr239/240)	CST	2434
α-tubulin	Santa Cruz	sc-23948
β-actin	Sigma Aldrich	A5441
GAPDH	CST	2118
